# DETECTION OF ELDER ABUSE BY FAMILY PHYSICIANS IN MONGOLIA: AN EXPLORATORY STUDY

**DOI:** 10.1093/geroni/igz038.2736

**Published:** 2019-11-08

**Authors:** Ariunsanaa Bagaajav

**Affiliations:** The Graduate Center, CUNY, New York, New York, United States

## Abstract

Elder abuse (EA) is a significant global social problem that jeopardizes the health and wellbeing of older adults around the world. Health providers can play a pivotal role in detection of EA and accessing resources and interventions. In Mongolia, where elder abuse is not widely recognized, family physicians (FPs) are particularly critical, providing frequent contact and home visits free of charge to community-dwelling older adults. However, little is known about FP knowledge and engagement with potential victims of EA. We interviewed 12 FPs from Ulaanbaatar participated via Skype about their knowledge of and responses to EA, using Grounded Theory data collection and analysis. All respondents reported encountering at least one case of EA in practice, and described the creative strategies they used to engage older patients, detect abuse, and prevent further harm. We present significant implications for guidelines for identification and prevention of EA in primary health care.

